# Looking at Biomolecular Interactions through the Lens of Correlated Fluorescence Microscopy and Optical Tweezers

**DOI:** 10.3390/ijms24032668

**Published:** 2023-01-31

**Authors:** Anahita Haghizadeh, Mariam Iftikhar, Shiba S. Dandpat, Trey Simpson

**Affiliations:** LUMICKS, Waltham, MA 02453, USA

**Keywords:** single-molecule biology, biophysics, optical tweezers, fluorescence microscopy, correlated force-fluorescence

## Abstract

Understanding complex biological events at the molecular level paves the path to determine mechanistic processes across the timescale necessary for breakthrough discoveries. While various conventional biophysical methods provide some information for understanding biological systems, they often lack a complete picture of the molecular-level details of such dynamic processes. Studies at the single-molecule level have emerged to provide crucial missing links to understanding complex and dynamic pathways in biological systems, which are often superseded by bulk biophysical and biochemical studies. Latest developments in techniques combining single-molecule manipulation tools such as optical tweezers and visualization tools such as fluorescence or label-free microscopy have enabled the investigation of complex and dynamic biomolecular interactions at the single-molecule level. In this review, we present recent advances using correlated single-molecule manipulation and visualization-based approaches to obtain a more advanced understanding of the pathways for fundamental biological processes, and how this combination technique is facilitating research in the dynamic single-molecule (DSM), cell biology, and nanomaterials fields.

## 1. Introduction

Biological systems go through complex and multicomponent events happening at the molecular level. These events establish key functions of biological systems. Precise observation of these molecular events provides a better understanding of the biological mechanisms and the behavior of biological systems. Techniques in structural biology (such as X-ray crystallography and cryo-electron microscopy (cryo-EM)) provide deep insights into the structure of the biomolecules at the atomic level as snapshots. While providing details into the structures of biomolecules such as nucleosomes to whole cells to viruses as well as subsequent molecular interactions, cryo-EM provides limited dynamic mechanistic information [1,2,3,4,5,6]. Other techniques such as nuclear magnetic resonance (NMR) methods delve into localized sub-nanosecond dynamics of molecules over a range of different time scales with atomic resolution in both solution and solid states [7]. However, NMR is limited to stable molecules and has an intrinsic low sensitivity, leaving gaps in mechanistic information [8]. On the other hand, techniques such as gel electrophoresis, surface plasmon resonance (SPR), and photoluminescence (PL) are considered bulk characterization methods, which often provide information on the time evolution of an “averaged” outcome of a multi-component system and lack details at the individual molecular level [9,10].

**Figure 1:**
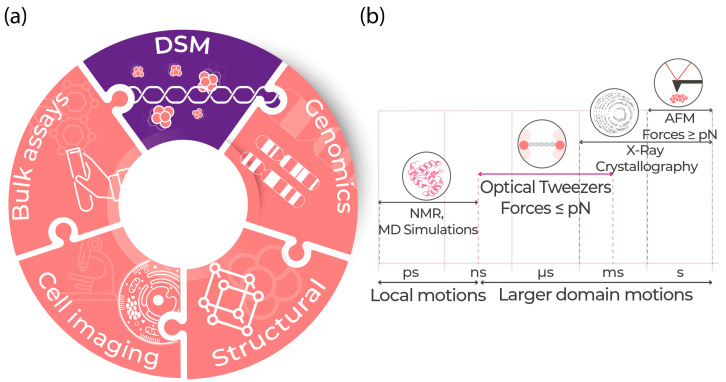
DSM Overview: (**a**) Overview of different domains of biology that together help in understanding the mechanism of actions at each step by determining structure, function, composition, and dynamic information. The field of dynamic single-molecule (DSM) technology aligns closely with other technologies by providing the missing pieces to the puzzle needed to fully understand biological processes (left). (**b**) Among well-known structure-function determination tools, DSM techniques using optical tweezers are positioned within the time scale (of μs to ms), where the majority of dynamic biomolecular events occur.

Single-molecule techniques have emerged as a powerful tool to bridge this gap toward a better understanding of biomolecular processes [9,10,11]. These techniques enable researchers to observe complex biological events, record their time evolution, and cast light on the molecular heterogeneity of individual components and their contributions to the overall process [9,10,11,12]. Single-molecule methods identify static and dynamic heterogeneity within the subpopulations in comparison with the bulk characterization methods [12]. Dynamic single-molecule (DSM) approaches are unique in understanding complex molecular interactions with ultrahigh sensitivity and precise temporal resolution, revolutionizing the field of molecular and cell biology. Adapting DSM research complements other well-established techniques to determine the structure, composition, and function of biological systems. DSM is a missing piece to the puzzle of obtaining a more complete picture of biological processes by providing insight into the dynamic and molecular heterogeneity, which leads to identifying mechanisms at the molecular level (Figure 1). 

Broadly, single-molecule experimental technologies can be divided into two categories: manipulation and visualization-based technologies [13]. Active manipulation of biological systems using the picoNewton-nanoNewton (pN-nN) range of forces at the single-molecule level is performed using methods such as optical tweezers, atomic force microscopy (AFM), magnetic tweezers, bio-membrane force probe, micro-needle manipulation, flow-induced stretching, and acoustic force spectroscopy (AFS), commonly known as force-based manipulation and force-based spectroscopy techniques [14,15,16,17]. Visualization-based single-molecule techniques primarily involve fluorescence-based visualization and detection of biological systems, most commonly using confocal, widefield (epifluorescence), or total internal reflection fluorescence (TIRF)-based microscopy [18,19]. Integrating a combination and correlation of these technologies has facilitated addressing the complexities of heterogeneous biomolecular interactions in multi-component systems [12,19]. The distinct advantage of these correlated DSM methods outweighs the complexity of building these instruments, establishing an easy-to-use workflow, and building a robust data analysis pipeline [12]. Many laboratories have worked towards building and developing unique single-molecule approaches, such as AFM and magnetic tweezers, that combine more than one of these techniques to increase the accessibility of these tools [20,21,22,23,24,25,26,27,28,29,30,31]. 

Atomic force microscopes are great force spectroscopy tools for studying various biological mechanisms such as inter and intramolecular interactions in near-physiological conditions [16,32,33]. Although AFM systems offer simple sample preparation and experimental setup for force spectroscopy, their higher measurable force resolution limits their application in the single-molecule field [34]. While measuring nonspecific interactions between the tip and random molecules can be challenging, recent developments such as a novel polymer nanoarray approach have enabled these measurements [16,35,36,37,38,39,40]. As one can expect, commercial AFM systems are easily accessible, but custom-built systems offer much higher control and flexibility to study biological processes. For example, high-speed AFM and the combination of AFM and fluorescence microscopy have been revolutionizing the single-molecule field since their emergence [34,41,42,43,44,45].

Magnetic tweezers (MT) are simple and robust single-molecule force spectroscopy tools that offer manipulation based on a magnetic field gradient [16,46]. MTs are the most straightforward tools for measurements at constant force (force clamp), parallel force measurements on multiple biomolecules, and 3D manipulation and rotation of biomolecules [29,46,47,48]. Unlike optical tweezers, MTs can exert force and torque in the nN ranges (F > 1nN) and perform measurements on hundreds of molecules simultaneously. MT force measurements and capabilities are based on a magnetic gradient; unlike optical tweezers, they do not cause photodamage and sample heating. However, their sensitivity is limited by video-based tracking, which hinders the detection of fast and small displacements [16,49,50]. An intriguing combination of magnetic and optical tweezers by R. Seidel et al. (2007) was used to study the supercoiled and torsional relaxed DNA dynamics under force [51]. Although recent improvements like the adaptation of reflection interference contrast microscopy (RICM) have improved the resolution of magnetic tweezers, they remain a robust tool for studying DNA-protein interactions, while optical tweezers cover a broader range of applications [46,52,53]. 

Compared to AFM and MT, optical tweezers provide a balance of sub-piconewton force measurements with high resolution and sensitivity, essential for biophysical characterization [54]. Optical tweezers, which rely on optically trapping micron-sized particles with a focused beam of light, were originally demonstrated by Arthur Ashkin [55,56]. While initially demonstrated for trapping atoms and dielectrics [56], optical tweezers garnered attention in the field of biophysics after their ability to trap and manipulate larger particles, such as viruses and bacteria, was observed [57]. Block et al. (1989) demonstrated the first quantitative measurement of forces in biological systems, such as in the flagella of bacteria [58]. Finer et al. (1994) used feedback-stabilized dual traps to measure the stepping of myosin on a single actin filament [59]. In one of the early applications of optical tweezers in DNA biophysics, Smith et al. (1996) demonstrated the stretching and elasticity of DNA molecules using dual-beam laser traps [60]. 

With advancements in optical tweezers, they have been combined with other single-molecule methods for biophysical studies. An early investigation by Ishijima et al. (1998) successfully combined dual optical traps with TIRF microscopy to observe individual ATP hydrolysis steps by a single myosin molecule on actin [61]. Lang et al. (2004) and Hohng et al. (2007) later reported on high-resolution optical tweezers with pinhole-based single-molecule fluorescence detection for investigating the unzipping and dynamics of DNA Holliday junctions [24,62]. Svedberg et al. (2006) and Dai et al. (2021) further combined optical tweezers with surface-enhanced Raman spectroscopy (SERS) for understanding aggregation behavior in nanoparticles and characterizing protein structures, respectively [63,64]. 

The high complementarity of force and fluorescence-based methods, enabling simultaneous manipulation and visualization for probing mechanical changes and biomolecular interactions, makes them a robust combination duo [65]. One such approach combines optical tweezers with confocal [66], STED [67], or a combination of widefield, TIRF, and label-free interference refraction microscopy (IRM) with a multichannel microfluidics system. These combinations unravel unique yet complex biomolecular processes such as DNA structure [68] and DNA-protein interactions [20,69], RNA structure and dynamics [70,71], protein structure and dynamics [72,73], biomolecular condensates and phase separation [74,75], cytoskeletal structures [76,77] and motor protein dynamics [77], and mechanobiology of cells [78,79].

This review highlights applications of correlated optical tweezers and fluorescence microscopy combined with microfluidic systems to understand key mechanisms of action for a broad range of biological systems. The most commonly studied mechanisms involve DNA–protein interactions, namely during DNA replication, DNA damage and repair, helicase activity, chromatin dynamics, and DNA editing using CRISPR-Cas-based systems. Next, we will focus on RNA and protein-based studies such as manipulating the structure of RNA and protein molecules and their key insights into the conformational dynamics of these folded biomolecules in the free-energy landscape. Further, we will investigate the cases where the material and rheological properties of disordered proteins forming biomolecular condensates and nucleic acid-protein co-condensates are studied using force spectroscopy. Moving from molecular interactions to macromolecular interactions, we will illustrate the roles and applications of correlative optical tweezers and fluorescence microscopy in understanding cytoskeletal structure and function, both in vitro and in vivo. In the future directions section, we will highlight new avenues and future outlooks of such combined approaches in new applications such as RNA biology, the characterization and handling of nanomaterials, and mechanobiology and cell-based measurements at a larger scale.

## 2. Discussion

### 2.1. DNA-Protein Interactions

**Figure 2:**
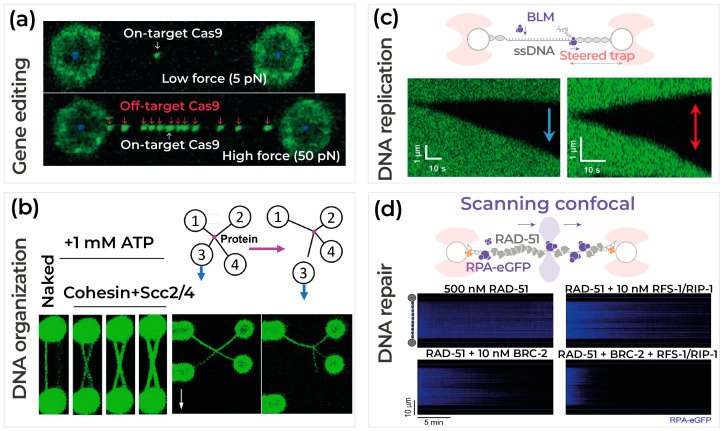
Understanding various DNA–protein interactions using correlated optical tweezers fluorescence microscopy. (**a**) Gene editing: a DNA molecule tethered between two optically trapped beads. The upper panel shows the on-target activity of the Cas9 protein when the DNA is held at a low force of 5 pN. Conversely, at a high force (50 pN), when the DNA molecule is stretched and under strain, several off-target activities appear, as shown with red arrows. (**b**) DNA organization: the left side depicts a cohesin bridge between two DNA molecules formed during the incubation of two DNA tethers in cohesin, ATP, and SCC2/4. The figure on the right shows how using 4 optically trapped beads can help in studying cohesin bridges between DNA molecules. Two DNA molecules were tethered between beads #1–2 and #3–4, respectively. Bead #3 was moved downward in the z-axis to apply force to the bridge (the blue and white arrows show this vertical movement) and measure its strength and sliding property. As shown, the cohesin tether keeps sliding on the DNA even after the exerted force breaks the high-affinity bond between the biotinylated DNA end and the streptavidin-coated bead. (**c**) DNA replication: Schematic of DNA unwinding mechanism by BLM helicase. The kymographs were formed when the fluorescence laser scanned the tethered DNA molecule continuously over 10 s. Sytox orange stain (green) was used to examine the dsDNA transition to ssDNA, where the absence of green fluorophore is an indicator of ssDNA. The left kymograph was acquired in the presence of 200 nM BLM at 30 pN, which showed unidirectional BLM’s DNA unwinding behavior. The right kymograph was obtained in the presence of 50 nM hRPA and BLM under the same amount of force (30 pN). (**d**) DNA repair: an experimental schematic where an optical tweezers-based single-molecule technique was used to resolve individual RAD-51 filament growth and measure their growth rates in replacing RPA-covered resected DNA in the HR repair mechanism. The impact of RFS-1/RIP-1 and BRC-2 on the RAD51 growth was shown in the kymographs. As depicted, RFS-1/RIP-1 has a stronger impact on the growth rate of individual RAD-51 nuclei in comparison with BRC-2. Figures are adapted with permission from reference [97], Copyright (2019) Springer Nature and open source references [82,90,92].

Many biological processes revolve around the interactions between protein machinery and DNA/RNA. DNA repair, DNA replications, gene editing and regulation of gene expression, and transcription are crucial to cells’ performance and depend on the interactions between proteins and DNA/RNA molecules [80,81,82]. Therefore, scientists have been trying to employ different techniques to better understand these interactions’ roles in cellular processes. The correlation of optical tweezer fluorescence imaging combined with microfluidics will provide the ability to manipulate and observe these interactions at the single-molecule level in controlled environments and in real-time. Here, we provide an overview of the DNA-protein interactions in four major processes of gene editing, DNA organization, replication, and repair using combined optical tweezers and fluorescence microscopy (Figure 2). In these studies, a DNA tether is usually formed using streptavidin-coated beads and biotinylated DNA ends. In other instances, accurate and stable site-specific attachment of DNA molecules to proteins is a requirement for many single-molecule force spectroscopy techniques. While maleimide chemistry is a common method for bioconjugation, Synakewicz et al. (2019) offer an alternative biorthogonal approach for site-specific conjugation using unnatural amino acids [83]. Integrated optical traps and microfluidics offer complete control over the DNA tethers as well as the biophysical and chemical stimuli. In addition, multicolor fluorescence imaging provides the ability to scan along the DNA length over time to observe the association and disassociations of differently labeled proteins, their genomic positions, and their lifetime on the DNA molecule [81,84].

### 2.2. Gene Editing 

In recent decades, RNA-guided gene editing techniques from bacterial defense mechanisms against viruses have been adapted for therapeutic purposes [85,86]. These techniques use clustered, regularly interspaced, short palindromic repeats and their associated proteins, known as CRISPR-Cas systems, to search, bind, and cleave a specific sequence of DNA. To realize CRISPR-Cas systems’ therapeutic potential, a variety of studies have been conducted to better understand their mechanisms and improve their specificities [86,87,88,89]. The CRISPR-Cas9 complex has been one of the most popular complexes in gene editing but has shown off-target activities under biologically relevant forces. Newton et al. (2021) used a combination of optical tweezers, microfluidics, and fluorescence microscopy to study these off-target activities at the single-molecule level [90,91]. A DNA tether was formed between two optically trapped beads, and Cas9 was introduced to the DNA by moving the DNA tether to a microfluidic channel containing the Cas9 proteins. As shown in Figure 2a, applying force, and stretching the DNA can expose the DNA and induce off-target binding of Cas proteins to the DNA [90]. Similar studies on Cas12, an alternative to Cas9 with fewer off-target activities, using correlative optical tweezers and fluorescence microscopy, revealed information about the searching and cleavage mechanisms of this complex. It was concluded that Cas12 complexes diffuse both randomly and bidirectionally to find their specific sequence target for the binding and cleavage mechanisms [88].

### 2.3. Chromatin Organization

Chromatin organization is a dynamic process that plays a key role in DNA repair, recombination, genome replication, and silencing [80,82]. Cohesin, a chromosome-associated protein, is a crucial participant in mediating cohesion between replicated sister chromatids and DNA packaging in chromatin fibers [81,84]. Gutierrez-Escribano et al. (2019) used correlated optical tweezers with fluorescence microscopy to investigate the “permanent and reversible” DNA bridges between two naked lambda DNA by cohesin in the presence of ATP and Scc2/4 [92]. Further force measurements using the optical tweezers revealed that reversible bridges can be disrupted at lower forces (5 pN < F < 40 pN). Whereas “permanent bridges” can slide on the DNA and resist extreme forces. This group also used four optically trapped beads to create intramolecular DNA bridges (Figure 2b). By moving bead #3 in the Y axis, they showed the cohesin tether keeps sliding on the DNA even after the exerted force breaks the high-affinity contact between the biotinylated DNA end and the streptavidin-coated bead [81]. 

### 2.4. DNA Replication

DNA replication includes major molecular events such as initiation, DNA unwinding, primer synthesis, and elongation, which are the result of precise collaboration between different proteins and dsDNA [80]. Correlative optical tweezers and fluorescence microscopy combined with microfluidics provide the means to study the replication process by tracking the interactions between different protein machinery with the DNA, step by step [81,82]. For example, Wasserman et al. (2019) used this combination to directly study the CMG63, a replicative helicase with a closed ring structure [84], and its interaction with DNA at the single-molecule level. Based on their correlated fluorescence and force microscopy-based measurements, it was revealed that the CMG ring can open its closed ring structure while transitioning from dsDNA to ssDNA [81]. Another study by Qin et al. (2020) using correlated optical tweezers and fluorescence microscopy uncovered the effect of human RPA (hRPA) proteins on the bloom syndrome protein, a DNA unwinding protein also known as BLM, and its DNA unwinding mechanism [82]. Using the integrated microfluidics system, the DNA unwinding process under different biological conditions such as force and concertation of BLM and hRPA was studied. As depicted in Figure 2c, it was shown that hRPA can promote the BLM’s DNA unwinding process from a nick and change the normal unidirectional behavior of the BLM’s unwinding process to two unwindings in the opposite directions from a nick on the DNA [82]. These results complement the previous studies using magnetic tweezers and single-molecule fluorescence imaging [93,94,95,96].

### 2.5. DNA Repair

DNAs are constantly exposed to endogenous and exogenous damaging agents [85]. To ensure cell health and function, DNA damage needs to be repaired instantly and accurately [86]. In addition to ensemble approaches, studying DNA damage, response, and repair at the single-molecule level using correlative optical tweezers and fluorescence microscopy has elucidated additional information about DNA damage recognition and different repair mechanisms and pathways [87,88,89]. In a study by Belan et al. (2021), a combination of optical tweezers, fluorescence imaging, and microfluidics was employed to understand the homologous recombination (HR) mechanism [97]. HR is a complex DNA double-strand break (DSB) repair mechanism and involves several steps and mediator proteins. The displacement of the RPA-coated ssDNA by RAD51 filaments, one of the crucial steps in HR DNA repair, was shown. In this study, a single lambda DNA was tethered between two optically trapped beads. ssDNA was generated by moving one bead from its original position and away from the second bead, stretching the DNA molecule under force. As one can expect, the ssDNA was coated by RPA (eGFP tagged in this report), ssDNA binding proteins. This process was observed in different biochemical environments, in the presence or absence of BRC-2 and/or RFS-1/RIP-1, using microfluidics. It was shown that including both BRC-2 and RFS-1/RIP-1 increases the RAD-51 filament growth rate on the RPA-coated ssDNA, which was associated with a reduction in the e-GFP signal in the recorded kymographs [90] (see Figure 2d). The same technique was successfully used to probe the molecular processes involving multi-protein complexes isolated from human cells’ nuclear extract [98].

## 3. RNA Structure and Function

RNA plays a key role as an information transfer intermediate between DNA and protein while regulating gene expression. The RNA coding family, mRNA, is responsible for protein synthesis inside the cell; the non-coding family of RNA plays an essential role in regulating downstream gene expression processes and controlling protein production. Similar to proteins, some non-coding RNAs are highly structured and undergo thermodynamic and kinetically controlled conformational changes in the folding free energy landscape [99,100,101]. RNA structures and their dynamics have been studied by various groups using the combination of two or more single-molecule techniques such as AFM, TIRF microscopy, confocal microscopy, optical and magnetic tweezers, and most recently using nanopore technology [100,102,103,104,105,106,107,108,109,110]. The combination of optical tweezers, microfluidics, and fluorescence microscopy has emerged as a great tool to understand the dynamics and functions of RNA structures. Moreover, this DSM technology has helped understand these structures and their functions through easy manipulation in the pN range and identifying the role of kinetically less stable RNA structures.

**Figure 3:**
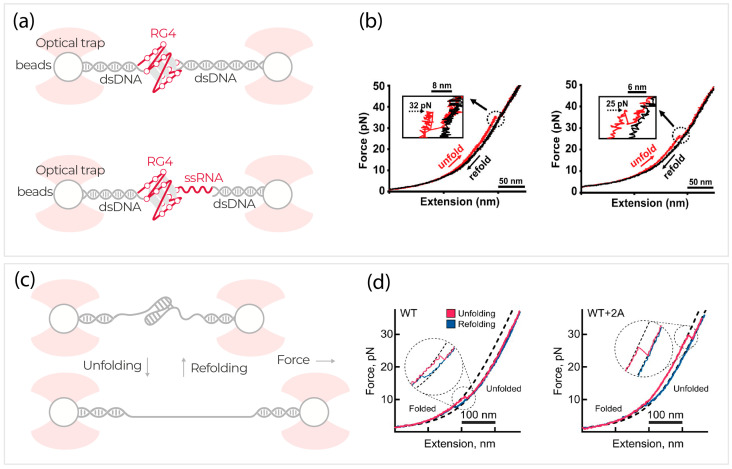
Overview of understanding various RNA structure dynamics using optical tweezers. (**a**) Schematic representation of RNA G-quadruplex (RG4) formed using single-stranded RNA (ssRNA) sequences without and with a proximal ssRNA extension, tethered between two optically trapped beads through double-stranded DNA. (**b**) Corresponding force-extension plots illustrate that the addition of a proximal ssRNA extension to RG4 weakens its mechanical stability by reducing the rupture force for the RG4. Adapted with permission from [53]. Copyright (2021) American Chemical Society. (**c**) Schematic representation of folded and unfolded RNA pseudoknot structures tethered between two optically trapped beads. (**d**) Corresponding force-extension plots illustrate that −1 PRF regulating protein 2A interacts with the RNA pseudoknot and increases its mechanical stability to promote −1 PRF controlled translation. Adapted with permission from reference [112] Copyright (2021) Springer Nature.

RNA G-quadruplex motifs (RG4) are a group of guanine (G)-rich RNA sequences that can form stacked G-quartets with monovalent cations between them, such as DNA G-quadruplex structures. RG4 is speculated to regulate key biological functions, such as transcription and translation regulation, RNP formation, and splicing regulation [101,111]. Whether these structures exist in the folded state in vivo is not clear yet and can be better understood by the structural polymorphism of the RG4 sequences. To understand the stability of an RG4 motif derived from a human telomere RNA, Ye et al. (2021) used optical tweezers alongside single-molecule Förster resonance energy transfer (smFRET) and nuclear magnetic resonance (NMR) [71]. An RG4 motif attached to DNA handles between two optically trapped beads remains stable at 100 mM Na^+^ concentration with a rupture force of ~32 pN (Figure 3). They showed that the presence of a proximal ssRNA extension following the RG4 can weaken the RG4’s mechanical stability by reducing the rupture force to ~25 pN. Using a supplementary endonuclease assay, the less stable RG4 structures can provide higher accessibility to G-rich sequences for machinery such as motor proteins [71]. 

RNA pseudoknots, another group of RNA structural elements, are specifically known for their functional role in RNA viruses [112]. RNA pseudoknots are often linked to regulating translation events, one such key event being programmed −1 ribosomal frameshifting (PRF) [112]. Essentially, RNA viruses use PRF as a translational control strategy, moderated by certain factors, to ensure the production of essential proteins for virus assembly and growth [70,112,113]. Hill et al. (2021) used optical tweezers correlated with fluorescence microscopy and microfluidics to study the 2A protein from the cardiovirus (EMCV) and its role in binding to the viral RNA pseudoknot and changing rupture forces [113]. Force extension curves revealed that 2A binding significantly favors the stabilization of folded RNA pseudoknots by increasing the average rupture forces by almost two folds compared to the viral RNA in the absence of 2A. Additional experiments showed that 2A binding stabilization of pseudoknot RNA selectively enables PRF translation during host cell infection [113]. 

Interestingly, while certain viral factors interact and influence viral RNA pseudoknots to increase viral infection, other antiviral proteins can reduce viral infection through a similar mechanism. Zimmer et al. (2021) identified a short isoform of the endogenous zinc-finger antiviral protein (ZAP-S) and showed that its overexpression and interaction with viral RNA pseudoknot can reduce the replication of the SARS-CoV-2 virus and hence its life cycle [70]. During SARS-CoV-2 infection, the viral RNA pseudoknot is shown to promote −1 PRF translation that leads to the formation of the viral RNA-dependent RNA polymerase (RdRP) necessary for viral replication. Using optical tweezers, Zimmer et al. (2021) showed that ZAP-S binding to the viral RNA pseudoknot prevents the structure from refolding into its native state and simultaneously leaves unfolding events unaffected [70]. In the absence of ZAP-S, the RNA pseudoknot structure unfolds under 15–20 pN, forcing an intermediate unfolding transition; however, it refolds back in two steps upon relaxation at ~11 pN. In the presence of ZAP-S, the unfolding behavior of the pseudoknot structure remained mostly unaffected, but strikingly, the refolding behavior was disrupted to return to the native folded state [70]. These studies collectively show that single-molecule pulling experiments unveil key features of RNA structures, conformations, and their role in regulatory mechanisms. 

## 4. Biomolecular Condensates Micro-Rheology and Manipulation

**Figure 4:**
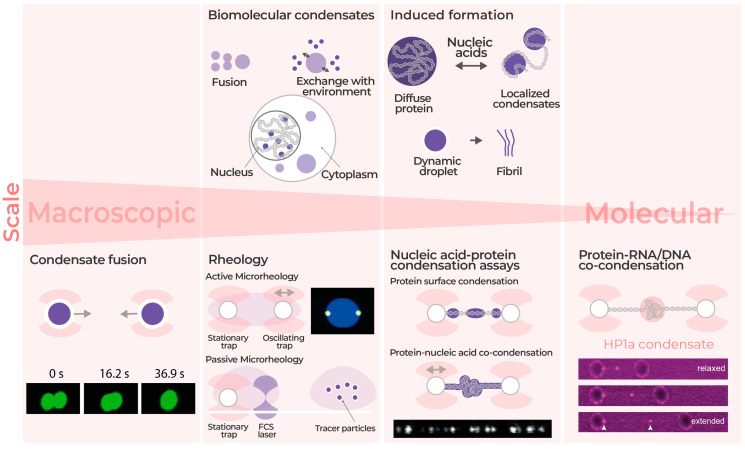
Phase Separation: Top row: An overview of biomolecule condensates, their structure, and the aging process. Bottom row, from left to right: Condensate fusion: how the fusion of biomolecule condensates is facilitated by trapping condensates directly in optical traps. An example of condensate fusion is depicted at the bottom left, where fusion speed is measured by observing the process in real-time. Rheology: Schematic diagram of active and passive rheology using two and one optically trapped beads inside the condensate, respectively. Schematic of single-molecule nucleic acid-protein condensation assays that can result in surface condensation or co-condensation. Surface condensation: The confocal image is from a study in which an optical tweezer set-up was employed to directly observe the condensation of Klf4-GFP on a DNA molecule tethered between two trapped beads. This study shows that the surface condensations on the DNA molecule form through a switch-like transition. Co-condensation: An example of the co-condensation of protein-RNA/DNA. Fluorescence confocal images were taken when the tethered DNA was in a relaxed (low force), intermediate, and extended (high force) state. This study showed these co-condensations resist high forces. The white arrowheads show HP1a-DNA condensates. Adapted with permission from [114] Copyright (2018) the American Physical Society, and open sources [74,132,133].

Cells are made of specialized compartments and organelles with specific functions and chemical compositions. These organelles are separated from the cytoplasm and other organelles by a membrane. However, there are other organelles that are membraneless. These organelles are formed and separated from their environment by liquid-liquid phase separation [79]. To understand the behavior of these membraneless organelles and their properties, scientists have been using biomolecular condensates, such as protein condensates, as a simpler model [114]. In addition to their value in understanding the membraneless organelles, these biomolecular condensates are home to many critical functions such as RNA metabolism, gene transcription and regulation, and stress response [115,116,117]. It is known that liquid-like condensates function as biochemical reaction centers, whereas more solid-like and elastic ones operate as structural units for the cell. Thus, it is crucial to characterize these condensates’ behavior and properties. Among the different techniques used in characterizing biomolecular condensates, correlated optical tweezers and fluorescence are beneficial because they provide the possibility of manipulating (trapping) and visualizing these condensates and droplets in real-time (Figure 4). In addition, optical tweezers with integrated microfluidics provide control over the temperature and biochemical environment for further studies on these condensates. Moreover, optical tweezers enable additional measurements such as fusion speed and frequency-dependent rheology of different biomolecule condensates [114,118]. 

Biomolecular condensates consist of structured proteins, intrinsically disordered and unstructured proteins, and RNA. Molecular condensates tend to coalesce together, and their speed of fusion can reveal information about their packaging density and liquidity [118,119]. It is known that the unstructured proteins pack loosely, and therefore droplets made of these proteins can fuse faster compared to the condensates made of structured proteins. Moreover, it is known that biomolecular condensates go through an aging process in which their density increases and functionality decreases over time, which leads to pathological implications [120,121]. Jawerth et al. (2020) showed that protein condensates have viscoelastic properties and follow the Maxwell fluid model, where their viscosity increases drastically compared to the elasticity modules during aging [74,122,123]. Thus, fusion speed can uncover information about both packing density and the age of the biomolecular condensates [118]. Macromolecular crowding, electrostatic interaction, and molecular flexibility are among other determining factors of fusion speed [74,114,123,124,125]. As mentioned above, optical tweezers (OT) can be used to trap and study biomolecular condensates individually. By decreasing the distance between the two trapped condensates, fusion can be observed in a controlled manner, and the exerted force can be measured at the same time [126]. The exerted force is a direct indicator of the material properties of the droplets. For example, the exerted force due to the fusion of two liquid-like droplets is smaller than the force exerted by the fusion of solid-like droplets [118]. Ghosh et al. (2021) used OT to measure the fusion forces of several types of condensates [127]. Intriguingly, they found the structural compactness of biomolecular condensate components as a new fusion speed determinant. Additional experiments showed a correlation between condensate aging and low fusion speeds [118].

Optical tweezers are great tools to study condensate material properties via active and passive rheology. In active rheology, two micron-sized beads are trapped inside a condensate. One bead is used to cause oscillatory movements inside the condensate, and the second bead is used to measure the condensate deformation caused by the oscillation. In passive rheology, fluctuations caused by the thermal energy of an optically trapped bead inside a condensate are measured over time [122,128,129]. Recent rheology-based measurements using optical tweezers show that biomolecular condensates exhibit viscoelastic properties and follow the Maxwell fluid model, with dominant elasticity and liquid-like responses in shorter timescales and longer time scales, respectively [122,127,130]. Through active rheology using optical tweezers, viscous and elastic modules can be measured. Active rheology by Ghosh et al. (2021) indicated that condensate fusion is regulated by shear stress relaxation [122,127].

It is known that condensates are present inside the nucleus and are involved in different processes such as DNA repair, transcription, and heterochromatin formation [131]. Even though biomolecular condensates have been studied in bulk, limited information is known about their interaction with other intercellular components such as single nucleic acid molecules [131]. Moving toward liquid–liquid phase separation on a smaller scale, at the single molecule level, heterochromatin Protein 1 (HP1) and forkhead box protein A1 (FoxA1) proteins have been observed to form condensates with DNA and chromatin [131,132]. During this process, also known as co-condensation, the HP1 protein binds to DNA and starts encapsulating the proximal fluctuations and compacting the DNA by exerting force on the non-condensed DNA to form mesoscale condensates [131,132]. Force measurements using optical tweezers show that these condensates can tolerate biologically relevant high external forces [132].

Another type of condensation that occurs between protein and DNA molecules in the nucleus is called surface condensation. Based on a study by Morin et al. (2022), measurements using optical tweezers show that the presence of DNA molecules can induce transcription factor Klf4 to form condensations at a much lower concentration than the required concentration for the bulk phase separation of Klf4 [133]. Further studies can elucidate the role of specific DNA sequences in initiating surface condensation [133,134]. Correlated optical tweezers and fluorescence microscopy combined with microfluidics provide control over the DNA molecule and protein concentrations. In addition to the ability to manipulate the DNA molecule during the protein–DNA co-condensation, these correlated techniques allow the observation of the process in real-time (Figure 4) For biomolecular condensations and phase-separated proteins, using correlated fluorescence optical tweezers provides insights at the single-molecule level into the dynamics of these biomolecules. [114,117,118,123,126,130,131,132,133].

## 5. Protein Dynamics

**Figure 5:**
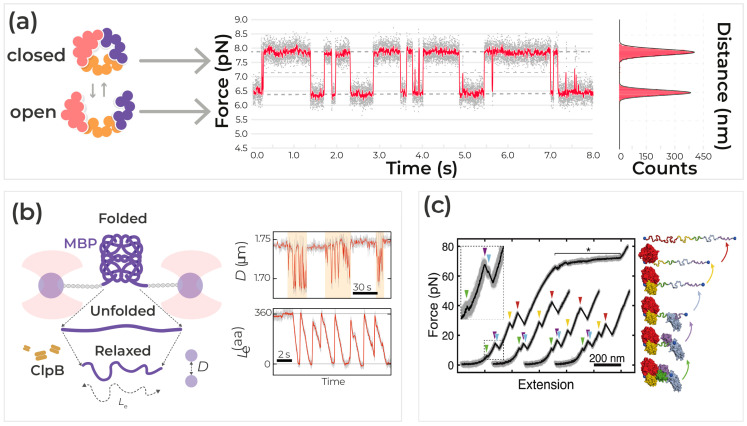
Protein Dynamics (**a**) Conformational states of example proteins moving from a closed to an open state correlated with fluctuations in force (pN range) over time (s). Each conformational state corresponds to a force level. Further on the right is a histogram denoting the events, or counts, of the protein in the closed state versus the open state. (**b**) ClpB is a processive translocase. This introduces the work of Sander Tans on the extrusion activity of a disaggregase. Hsp100 disaggregases such as ClpB and Hsp104 are proposed to catalyze this reaction by translocating polypeptide loops through their central pore. This model of disaggregation is appealing, as it could explain how polypeptides entangled within aggregates can be extracted and subsequently refolded with the assistance of Hsp70. However, the model is also controversial, as the necessary motor activity has not been identified. (**c**) Maciuba et al. illustrate representative force extension curves (FECs) from the first pulls of elongation factor-G (EF-G). Overstretching of the DNA handles (asterisk) is shown only for the first trace. All traces display the characteristic unfolding pattern of the five-domain protein (arrowheads, color-coded by domain). The inset shows a magnification of the low-force region from the first FEC. Figures are adapted with permission from reference [141] Copyright (2020) Springer Nature, and reference [72] Copyright (2021), Springer Nature.

Proteins play essential roles in all life processes, from accelerating various metabolic reactions to controlling signaling transduction. The regulatory and catalytic functions of enzymes require dynamic changes in protein structure [135,136]. The interplay between molecular motions and functions has been extensively studied and reveals a wide range of motion distances (Å to nm) and timescales (ps to s) [135]. In many cases, the exchange between different functional states for the enzymes occurs on a µs-ms timescale, which also coincides with typical timescales of protein folding [137]. The ability to resolve in real-time the dynamic and kinetic behavior of proteins at relevant timescales provides an opportunity to understand the intricate and complex behavior of proteins as they perform their processes. New methodological approaches focus more on determining structural fluctuations and their consequences on the function of proteins than just the static three-dimensional structures (Figure 5a). 

Sophisticated biophysical methods better elucidate the mechanisms of various biological processes. Emerging technologies give an understanding of protein variability, time-dependent variability (dynamic disorder), and the innate mechanistic behavior of an enzyme during individual binding, unbinding, or catalytic events. In addition to spatial arrangement, dynamic fluctuations are a driving force for several types of molecular interactions. In particular, the Rief laboratory pioneered protein nanomechanics with single-molecule fluorescence accuracy using GFP protein as a model system [138,139,140]. The research illustrated the vast capabilities of studying specific protein dynamics at the single-molecule level. To determine the accurate mechanism for the translocation of polypeptide loops in Hsp100 disaggregases, Avellaneda et al. (2020) used optical tweezers with fluorescence [141]. By trapping and manipulating maltose-binding protein (MBP) with optical tweezers (Figure 5b), a force can be applied to prevent spontaneous refolding [141]. Then, the addition of the disaggregase ClpB and ATP would result in the refolding of MBP in brief bursts of contractions, as shown in Figure 5b [141]. Further experiments with confocal imaging correlated with the optical tweezer manipulation detected movements of certain polypeptide loops when ClpB was bound, thus elucidating the mechanism of action for ClpB.

Accurate characterization of dynamic travel time between enzymatic states is important for understanding protein function. Protein dynamics are characterized not only by the timescale of the fluctuations (a kinetic component) but also by the amplitude and the directionality of the fluctuations (a structural component). Consequently, the energy landscape representing a protein, which has numerous atoms, is highly multi-dimensional. Optical tweezers enable precise manipulation of biomolecules while simultaneously monitoring their response to force and displacement with nm resolution. For example, Maciuba et al. (2021) demonstrate a streamlined approach for investigating complex molecular assemblies with single-molecule force spectroscopy with optical tweezers [72]. Developing a workflow for cell-free protein synthesis, allowing for the study of unstable or intrinsically disordered proteins [72]. In this paper (Figure 5c), researchers utilized the SpyTag/SpyCatcher system to tether the multidomain protein EF-G [72]. As the force applied to EF-G increases, the protein unfolds its five domains step by step, as shown by the force curve in Figure 5c [72]. Another study by Moessmer et al. (2022), using high-resolution optical tweezer data, demonstrates in real time how the glucocorticoid receptor is completely unfolded by the Hsp70/Hsp40 chaperone system [142,143]. This chaperone complex stabilizes new unfolding intermediates, allowing Hsp70/40 to directly interact with the folded core of the protein and unfold them upon ATP hydrolysis [142,144].

Overall, the intricate manipulation and monitoring provided by single-molecule force spectroscopy illuminate protein folding mechanisms such as the GroEL-ES chaperone-assisted folding mechanism, in which enhanced collapse plays a vital role. This proposed unfolding model was directly observed through single-molecule optical tweezer experiments and further facilitates an understanding of the folding mechanism, which is distinct from current models [73]. Delving even further into the source of protein folding, Wruck et al. (2021) combine optical tweezers and single-molecule FRET (fluorescence resonance energy transfer) to observe proteins as they are translated into the ribosome [145]. Researchers found that the ribosomal tunnel accelerates the folding of the small zinc-finger domain (ADR1a) and stabilizes the folded state, a similar effect to chaperones [145]. In addition to protein folding, directly monitoring protein complex formation is a key link for understanding functions such as molecular chaperones [146], intrinsically disordered protein networks [147], DNA- and RNA-binding proteins [148,149], including novel homologs of the CRISPR-Cas9 complex [150], tumor repressors [151], and steroid receptors [152], among many other systems. For example, Zimmer et al. (2021) demonstrated that the zinc-finger antiviral protein (ZAP-S) directly interacts with the SARS-CoV-2 RNA to interfere with RNA folding [70]. 

## 6. Cellular Structure and Transport Processes

The cytoskeleton is a critical cell component that regulates cell shape, cell migration, and intracellular organization [153]. It consists primarily of microtubules, intermediate filaments (IFs), and actin filaments, together with motor proteins and crosslinkers, each of which plays unique roles and contributes to the cytoskeleton’s diverse functionality. Alterations in these cytoskeletal components can cause a variety of diseases, ranging from neurological disorders to muscular dystrophies, highlighting a need to better understand the cytoskeleton [153]. Currently, separate tools exist for understanding either cytoskeletal dynamics or mechanics. Simultaneously studying the organization, dynamics, and mechanics of filaments and the interaction of filaments with force-generating motor proteins is crucial to fully understand the mechanisms involved in emergent cellular processes and associated pathophysiologies [153,154]. Therefore, researchers seek a comprehensive tool that can directly correlate intramolecular dynamics with mechanics.

**Figure 6:**
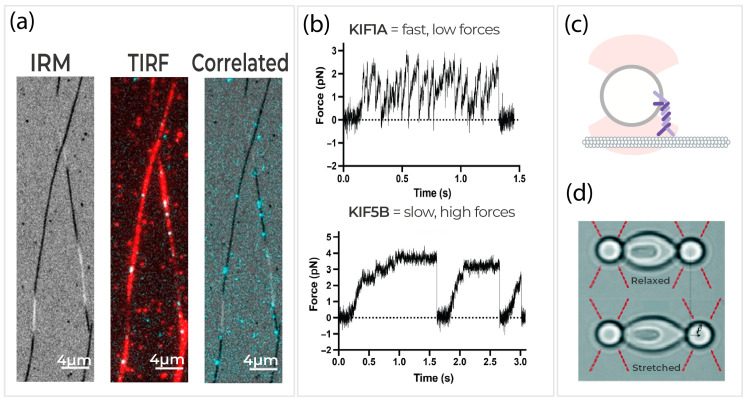
Characterizing the biophysical properties of motor proteins. (**a**) Example images of IRM: unlabeled microtubules visualized with IRM (note: microtubules near the surface are dark while non-adherent regions are lighter), TIRF: overlayed microtubules (red, labeled with Hilyte647) with GFP-labeled kinesins (cyan), and Correlated: overlay of IRM images with GFP-labeled kinesins. (**b**) Representative force versus time records of bead movement driven by single molecules of WT KIF1A and WT KIF5B. (**c**) Illustration of a standard surface assay for studying cytoskeletal motor proteins, such as kinesins, with optical tweezers. (**d**) Shows a red blood cell confined between two optically trapped beads in a relaxed and stretched state. In this study, the effects of strain rate and atorvastatin on RBC’s mechanical properties were measured by optical tweezers. Figures are adapted with permission from reference [77] Copyright (2021) Rockefeller University Press and reference [78] Copyright (2022) Royal Society of Chemistry.

Using a single-molecule approach enables the simultaneous study of the dynamics and mechanics of cytoskeletal processes (Figure 6a). Specifically, correlated force and displacement measurements with fluorescence (total internal reflection fluorescence, TIRF) and label-free (interference reflection microscopy, IRM) microscopy achieve an unparalleled look at the cytoskeleton with single-molecule resolution. At the molecular level of the cytoskeleton, the step sizes (~10 nm) [155], dynamics (~100 Hz) [84], and forces (~10 pN) [156] are the standard, thus limiting researchers in studying its biological properties. Imaging techniques coupled with force manipulation allow the visualization of filaments and motor proteins with good spatial resolution and the mechanical forces at work, enabling researchers to track and analyze their activity. For instance, single-molecule force spectroscopy techniques such as optical tweezers measure molecular activity and mechanical forces at rates in the kilo-to-mega Hz range that cannot be determined from any other technique. Budaitis et al. (2021) demonstrated that pathogenic mutations in the kinesin motor protein stall at forces of ~3 pN but more frequently detach at lower forces (Figure 6c,d) [77,157]. 

Using correlative IRM/TIRF technology, microtubules were imaged on the surface using IRM, while the motility of GFP-labeled kinesin motors on microtubules was measured using TIRF (Figure 6b). These images show how correlative imaging techniques enable the study of dynamic cytoskeletal interactions at the single-molecule level with sharp contrast and spatial resolution. It is important to note that, thanks to the label-free capacity of IRM microscopy, there is no need to label the microtubules.

A deep study of vimentin, a typical IF protein, using optical tweezers reveals crucial loading rate dependence of mechanical response, which further implies that IFs could serve to protect eukaryotic cells [158]. Further experiments demonstrate that the strain-induced unfolding of B-sheets introduces a third conformational state [76,159]. In addition, researchers better understand the IF dependence on pH and ionic strength on mechanical stability [160,161]. Overall, the unique capabilities of single-molecule force spectroscopy allow for the vast determination of both the biophysical and mechanical properties of the cytoskeletal system. 

Sorkin et al. (2020) illustrated the flexibility and power of a system coupling optical tweezers with imaging capabilities provided when investigating Synaptotagmin-1 (Syt1), a calcium sensor protein, induced membrane remodeling [162]. Researchers directly prove that Syt1 binds significantly more strongly to membranes than to other Syt1 molecules, resolving the controversy involving its mode of action during synaptic vesicle fusion [162]. Direct observation of protein binding events and membrane interactions is critical for the understanding of the cytoskeletal system. For example, Siahaan et al. (2022) demonstrate that Tau protein molecules can form cohesive envelopes around microtubules [163]. In particular, Tau envelopes form cooperatively and locally alter the spacing of tubulin dimers within the microtubule lattice. Furthermore, Tau could potentially divide the microtubule surface into functionally distinct regions, some areas biased for motor protein movement [160]. 

## 7. Future Directions

Correlated force and fluorescence single-molecule approaches have emerged as great tools, not only for biophysicists but also for biologists and physicists, in understanding the missing details of structure-function relationships and materials’ properties. For example, employing correlated force-fluorescence approaches to look at biophysical and material properties of biomolecular condensates and measuring their micro-rheology has shown new possibilities for studying and understanding condensate properties. Similarly, looking at DNA-protein interaction through simultaneous manipulation and visualization in real-time has established new ways to understand complex, multi-component processes such as DNA replication, organization, damage repair, and editing. The adaptation of dynamic single-molecule approaches is increasingly benefiting various applications in the biology field. Thus, new generations of applications are anticipated, where the implementation of correlated force and fluorescence microscopy can aid in understanding complex biological processes and nanomaterial handling.

### 7.1. RNA Biology

RNA biology is a research area of increasing interest, where researchers are focused on understanding the structure-function relationships of different forms of RNA. In the past couple of decades, researchers have used optical tweezers and single-molecule fluorescence microscopy separately to understand different types of RNA structures (such as hairpins, ribozymes, and riboswitches) and their related functions (such as transcription, translation, and splicing) [54,99,106]. RNA-related machinery, such as RNA polymerases, and ribosomes, is known to act as molecular motors that exert sub-piconewton range forces while in action. Employing tools such as optical tweezers is helpful to probe these molecular mechanisms at the single-molecule level [26,164,165,166,167,168].

In earlier sections, we discussed the adaptation of correlated force and fluorescence techniques for studying RNA structures at different forces. Just like proteins, conformational dynamics and long-range interactions of regulatory RNA elements such as hairpins, riboswitches, and ribozymes can be studied with optical tweezers in correlation with smFRET. Duesterberg et al. (2015) reported correlated force-smFRET studies to measure secondary and tertiary structural changes in a TPP riboswitch during folding and ligand binding [169]. RNA folding pathways during gene regulation, the existence of short-lived metastable states, and long-range interactions can all be accurately identified through a correlated force-fluorescence-based approach. 

The combination of optical tweezers and fluorescence microscopy is a perfect tool for understanding RNA elements far beyond just their structures. For example, the transcription cycle in prokaryotes happens in two key steps: (1) initiation, where RNA polymerase recognizes the promoter site on DNA, binds to the DNA, and then unzips the DNA to form a bubble while recognizing the template strand; (2) elongation and termination, where elongating RNA polymerase scans over the DNA to decode the template into mRNA, followed by termination, often induced by certain factors, to signal the end of mRNA synthesis and release [167]. Many research groups have used single-molecule force-based assays (including optical tweezers) to determine how RNA polymerase identifies a promoter, initiates transcription, elongates and pauses along the DNA template, proofreads for errors, and terminates transcription [168,170,171,172]. Adding fluorescence detection (fluorescence colocalization, smFRET, etc.) would provide improved insights into processes such as the co-transcriptional folding of RNA, the role of transcription factors, and proofreading by RNAP.

Similar to transcription, the translation process uses templates of coding RNA (mRNA) to make proteins essential for gene regulation. Each of these translation steps involves template mRNA, ribosomal subunits, tRNAs, and a variety of factors that have been extensively studied using a wide range of single-molecule techniques, showing the precise kinetic control of each component and forces generated by the ribosome during this process [167,173,174,175,176,177]. However, to understand the translation mechanism better, many questions need to be answered, and that is where a correlated force-fluorescence approach can add value. Desai et al. (2019) used optical tweezers with single-molecule fluorescence detection to understand the role of mRNA hairpins in determining the translocation kinetics of ribosomes [178]. This technique can be used to investigate unique translational mechanisms such as co-translational protein folding [145] and transcription-translation coupling [179].

### 7.2. Nanomaterials

Although electron microscopy techniques such as SEM and TEM are ideal tools to study nanomaterials, optical tweezers can be used for the manipulation and precise placement of nanoparticles such as semiconductor nanowires to assemble complex circuits and lab-on-a-chip devices [180,181,182]. In addition, using optical tweezers, the interaction between single nanoparticles can be studied at high temporal resolution. The correlation between optical tweezers and fluorescence microscopy will be beneficial in the characterization of nanomaterials at the single-particle level.

Raman spectroscopy uncovers the structural fingerprint of materials according to their vibrational modes. Metallic nanostructures in bulk have been used to intensify the typically weak Raman signal through their plasmonic effects [183]. Dai et al. (2021) reported that the combination of Raman spectroscopy and optical tweezers can be used to develop surface-enhanced Raman spectroscopy (SERS) with single-molecule level sensitivity [63]. In this work, two silica beads coated with silver nanoparticles were optically trapped to generate tunable plasmonic enhancement fields, known as hotspots, for the Raman probe to identify proteins’ structures in diluted solutions [63]. 

### 7.3. Mechanobiology

At larger scales, correlated fluorescence microscopy and optical tweezers have a variety of applications in mechanobiology and nanomaterial handling and characterization [184]. Optically trapped beads can be used to accurately manipulate single cells and measure their response to biochemical and biophysical stimuli, membrane deformation, or intercellular interactions. For example, forces during phagocytosis and filopodia formation and protrusion can be measured accurately with simultaneous visualization of the interactions and movements in real-time using fluorescence microscopy [185]. Optical tweezers have been used to study adherent cells where physical stimulation from the surface is present as well as isolated and suspended single cells in solutions to eliminate surface adhesion and cell-cell interactions [78,186,187]. The combination of microfluidics, optical tweezers, and fluorescence microscopy enables researchers to study the mechanical properties of single cells in physiologically relevant conditions as well as uncover the impact of different biochemical stimuli on these properties [186,187]. For example, the mechanical properties of red blood cells (RBC) before and after treatment with atorvastatin were studied using optical tweezers. In this study, single RBCs were confined between two optically trapped beads. Stiffness measurements at different rates confirmed that atorvastatin alters the mechanical property and softens the red blood cells [78]. Other measurements, such as the strength and location of ligand-receptor pairs on single cells, are also possible [188].

At smaller scales, several labs have been using home-built and commercial optical tweezer systems with integrated microfluidic devices to investigate the behavior of bacteria for microbiology and biofilm studies. For example, bacteria’s response to chemical stimuli has been extensively studied based on their swimming behavior using optical tweezer systems. Optimal experimental parameters have been identified to decrease the IR radiation damage and increase the “killing time.” Moreover, it is shown that oscillating and dual-beam optical tweezers enable the horizontal alignment of bacteria, which is important for simultaneous manipulation and observation [189,190,191,192,193,194,195].

## 8. Conclusions

Although optical traps remain one of the most versatile force measurement and manipulation tools, they have drawbacks that must be considered during different measurements. As optical tweezers work like a Hookean spring, force measurements’ resolution depends on the accuracy of the calibration for obtaining the trap stiffness (spring constant) [196,197,198,199]. Thus, high-resolution force measurements are limited to optically homogenous samples. Due to challenging calibration in heterogeneous environments, until recently, force measurements inside heterogeneous media have been more relative than quantitative [199,200,201,202,203]. Besides the purity and homogeneity of samples, the concentration of samples should be minimal and optimized to avoid trapping more than one particle/molecule at a time [196]. Another important fact is that objectives with a high numerical aperture (NA) and a laser power of ~10^9^–10^12^ W/cm are used to create optical traps with high stiffness. This can lead to local heating at the focal point, which can cause damage to the sample and alter temperature-dependent and enzymatic reactions [204,205,206]. Despite these drawbacks, dynamic single-molecule techniques such as correlated optical tweezer-fluorescence microscopy tools have enabled researchers to investigate the dynamic behaviors of biomolecules and understand key mechanisms at the single-molecule level. In this review, we outlined several applications of correlated optical tweezer-fluorescence microscopy, using homebuilt and commercial systems, for understanding a wide range of biological mechanisms involving DNA-protein interactions, protein and RNA folding, biomolecular condensates, and mechanobiology at high temporal resolution (in milliseconds) and sensitivity (sub pN). These studies exemplify a wide range of biological processes whose molecular mechanisms can be better understood at the single-molecule level. We also illustrated the role of manipulation at the molecular level (using sub-pN range force) to understand certain biologically relevant processes and allow manipulation of the molecule of interest as required during certain biological events. We also discussed how optical tweezers can be used to understand the material and rheological properties of biomolecular condensates. The examples discussed here present a clear case for the application of correlated optical tweezers fluorescence microscopy techniques in a broad range of biological systems. Integrating multi-channel microfluidics and strategic assay designs into the core technology has facilitated a comprehensive and well-controlled workflow for advanced manipulation, observation, and investigation of complex biological systems. 

There are still many challenges in this field that need to be addressed to make such correlated technologies applicable to broader applications. A key limitation of optical tweezers is their low throughput [54]. OT observations can provide detailed insights at high temporal resolutions, limited by detector efficiency and short-lived conformational states that often get overlooked with other single-molecule techniques (such as magnetic tweezers and TIRF microscopy). However, such high-resolution observations of each trapped molecule limit the number of molecules observed in a given time. Low throughput can also be due to workflow limitations with optical tweezers assays, such as “fishing” for the right molecule between the traps. During the “fishing” step, one of the trapped beads is moved back and forth to form a tether with the target molecule attached to the other bead fixed in a trap. While an optimized concentration of target molecules and the tethering chemistry between molecules and beads can increase tethering efficiency and reduce “fishing” time, it can certainly be a limiting step for studying high-throughput assays with optical tweezers. Technological advancements such as parallelizing optical traps to generate an array of traps through timesharing or holographic optical tweezers can address the challenges of lower throughput with optical tweezers [207,208]. Another way to improve the throughput and save time during limiting and repetitive steps can be through experimental automation, which allows users to control and automate workflows, increasing accuracy, efficiency, and throughput for the research experiments [66]. Irrespective of the challenges, correlated technologies, including optical tweezers and fluorescence microscopy, serve a myriad of applications. 

More recently, such technologies are getting adopted far beyond the single-molecule level. As we discussed in some cases in the mechanobiology sub-section, correlated tools are increasingly being adopted to detect, measure, and visualize molecular interactions at the single-cell level. The ability to measure lateral and axial forces on individual cells has helped researchers reveal mechanical complexities in a wide variety of cell-based systems, such as RBC [185] and monocytes [181]. Standardized force calibration methods, integrated cell-based microfluidics, integrated widefield and label-free imaging techniques, and the development of robust workflows would enable such technologies to better elucidate cell-mechanics processes and bridge the gap between in-vivo and in-vitro studies. Such developments combined will have a greater impact toward a better understanding of molecular and cellular forces and interactions, which could be fundamental causes for diseases, drug discovery, and therapeutics.

## Data Availability

Not applicable.
